# Bevacizumab-based first-line chemotherapy in elderly patients with metastatic colorectal cancer: an individual patient data based meta-analysis

**DOI:** 10.18632/oncotarget.23475

**Published:** 2017-12-20

**Authors:** Christine Koch, Anna M. Schwing, Eva Herrmann, Markus Borner, Eduardo Diaz-Rubio, Efrat Dotan, Jaime Feliu, Natsuko Okita, John Souglakos, Hendrik T. Arkenau, Rainer Porschen, Miriam Koopman, Cornelis J.A. Punt, Aimery de Gramont, Christophe Tournigand, Stefan Zeuzem, Joerg Trojan

**Affiliations:** ^1^ Department of Gastroenterology, University Liver and Cancer Centre, Frankfurt, Germany; ^2^ Institute of Biostatistics and Mathematical Modelling, Johann Wolfgang Goethe-University, Frankfurt, Germany; ^3^ Medical Oncology Institute, Inselspital, Bern, Switzerland; ^4^ Department of Oncology, Hospital Clínico San Carlos, Madrid, Spain; ^5^ Fox Chase Cancer Center, Philadelphia, PA, USA; ^6^ Department of Medical Oncology, La Paz University Hospital, CIBERONC, Madrid, Spain; ^7^ National Cancer Center, Tokyo, Japan; ^8^ Department of Medical Oncology, University Hospital of Heraklion, University of Crete, Crete, Greece; ^9^ Sarah Cannon Research Institute, London, UK; ^10^ Klinikum Bremen Ost, Bremen, Germany; ^11^ Department of Medical Oncology, University Medical Centre, Utrecht, The Netherlands; ^12^ Department of Medical Oncology, Academic Medical Center, University of Amsterdam, Amsterdam, The Netherlands; ^13^ L'Institut Hospitalier Franco-Britannique, Paris, France; ^14^ University of Paris Est Creteil, Henri-Mondor Hospital, Créteil, France

**Keywords:** metastatic colorectal cancer, elderly, first-line chemotherapy, bevacizumab

## Abstract

**Background:**

The aim of this meta-analysis was to evaluate efficacy and safety of first-line chemotherapy with or without a monoclonal antibody in elderly patients (≥ 70 years) with metastatic colorectal cancer (mCRC), since they are frequently underrepresented in clinical trials.

**Results:**

Individual data from 10 studies were included. From a total of 3271 patients, 604 patients (18%) were ≥ 70 years (median 73 years, range 70–88). Of these, 335 patients were treated with a bevacizumab-based first-line regimen and 265 were treated with chemotherapy only. The median PFS was 8.2 vs. 6.5 months and the median OS was 16.7 vs. 13.0 months in patients treated with and without bevacizumab, respectively. The safety profile of bevacizumab in combination with first-line chemotherapy did not differ from published clinical trials.

**Materials and Methods:**

PubMed and Cochrane Library searches were performed on 29 April 2013 and studies published to this date were included. Authors were contacted to request progression-free survival (PFS), overall survival (OS) data, patient data on treatment regimens, age, sex and potential signs of toxicity in patients ≥ 70 years of age.

**Conclusions:**

This meta-analysis suggests that the addition of bevacizumab to standard first-line chemotherapy improves clinical outcome in elderly patients with mCRC and is well tolerated.

## INTRODUCTION

Colorectal cancer (CRC) is one of the most common cancers worldwide with over 1,300,000 new cases each year [1]. The incidence rate of colorectal cancer increases with age, rising from 8.4 per 100,000 at age 40–44 years to 127.8 per 100,000 at age 70–74 years and 196.2 per 100,000 at ≥ 75 years [1]. In Europe alone, over 300,000 new patients are diagnosed annually [1] and more than 61% of these patients are ≥ 70 years of age [2].

Fluoropyrimidines, irinotecan and oxaliplatin are the standard cytotoxic agents used in the treatment of metastatic colorectal cancer (mCRC). Survival benefit with standard chemotherapy regimens has been shown to be similar for mCRC patients ≥ 70 years of age compared with those < 70 years, and there is no marked difference in tolerability profiles [3].

Since elderly patients are more likely to suffer from comorbidities and to present with age-related decline in organ function (especially liver, kidney and bone marrow) than younger patients, there is an under-representation of elderly patients in cancer treatment trials, with < 10% of patients enrolled in colorectal cancer clinical trials being > 70 years of age [4–6]. For CRC trials, the median age of patients is 63 years, while the median age of patients at diagnosis of CRC is 72 years [1]. As a result of this, findings from colorectal clinical trials do not necessarily fully reflect real-life experience as the proportion of elderly patients is low and any subgroup analysis evaluating treatment efficacy in elderly patients from a single trial is difficult to perform with sufficient power because of this low number of patients. One phase III clinical trial that evaluated bevacizumab plus capecitabine versus capecitabine alone elderly patients with previously untreated mCRC was the AVEX trial [7], which found the bevacizumab plus capecitabine combination to be effective and well tolerated. Apart from AVEX, no further randomized studies addressing this sometimes more fragile population are available. Besides, some small cohort or single-arm studies enrolling elderly patients suggested that these patients also benefit from the addition of bevacizumab to standard chemotherapy [8, 9]. Meta-analysis of a number of similar clinical trials is an option to allow analysis of a sufficient amount of clinical data.

Treatment guidelines can vary considerably (go/go slow/no go) for elderly patients, and comprehensive geriatric assessment is rarely implemented in clinical practice [10, 11]. Therefore, because of the relative lack of clinical data in elderly patients with CRC, clinical decision making is driven by the assumed outcome and the assumed safety and tolerability.

The efficacy of standard chemotherapy regimens can be further improved with either the combination with bevacizumab, a humanized monoclonal antibody against vascular endothelial growth factor (VEGF), or with the epithelial growth factor receptor antibodies cetuximab or panitumumab in patients with RAS wild-type tumors [12]. As a result of the benefits observed with these regimens in controlled clinical trials, it is of critical importance to determine how the elderly mCRC population responds to and tolerates such treatment regimens.

The aim of this meta-analysis was to combine data from a number of clinical trials to evaluate the efficacy and safety of first-line chemotherapy with or without a monoclonal antibody in elderly patients (≥ 70 years of age) with mCRC.

## RESULTS

### Literature search results

**Figure 1:**
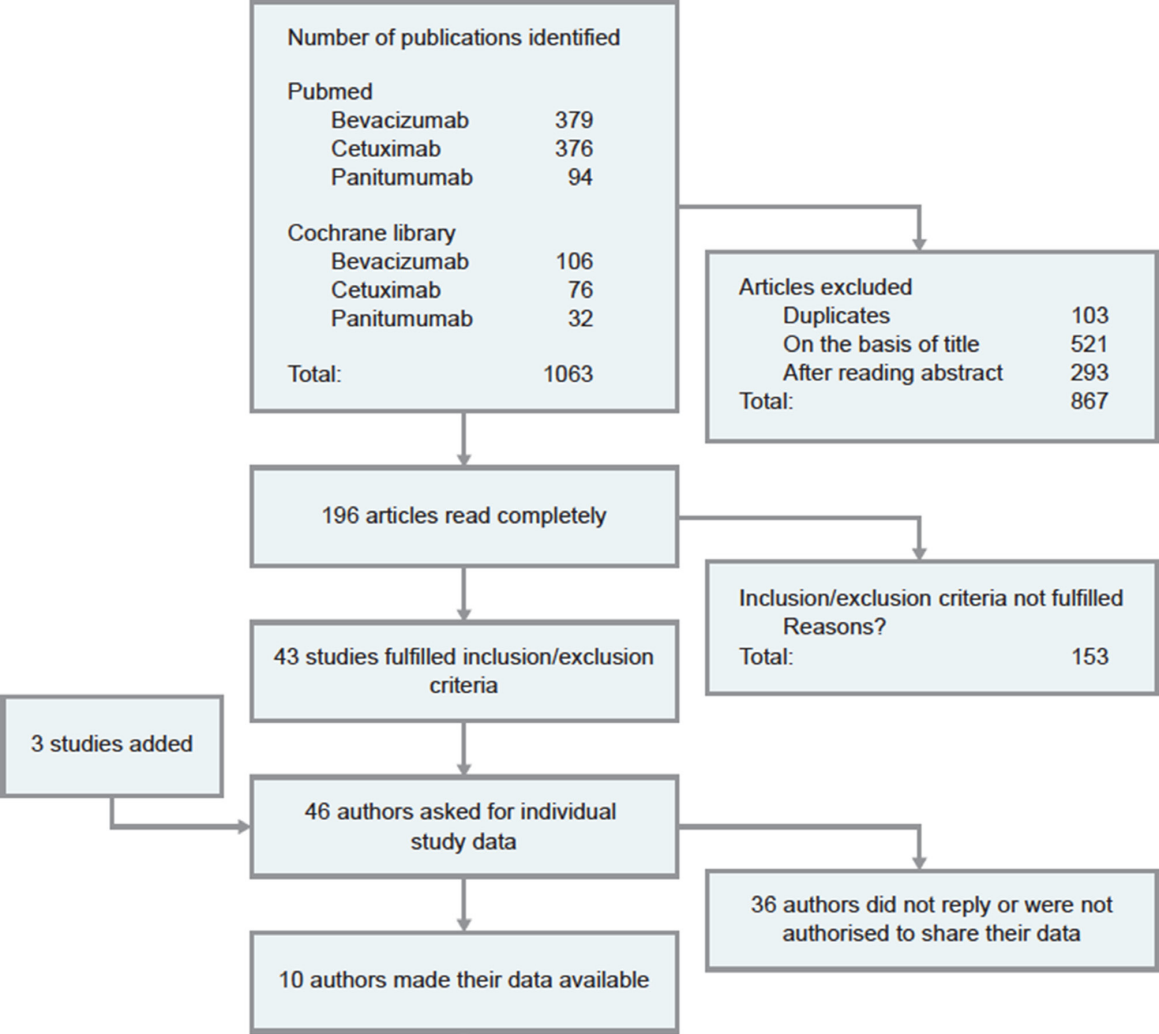
CONSORT flow diagram

Overall, 1063 potential publications were identified on the PubMed and Cochrane databases; of these, 867 were excluded based on reading the title, the abstract or being duplicates, leaving 196 articles to be read completely. Of these 196 articles, 43 fulfilled the inclusion and exclusion criteria and 3 further studies were identified; hence, 46 authors and three pharmaceutical companies were contacted for individual study data. In total, we received primary data from 10 studies [13–22] with different chemotherapeutic agents or regimens, all of them were from cooperative groups only. The chemotherapy backbone included infusional 5FU or capecitabine based combination regimens with either oxaliplatin or irinotecan ± bevacizumab (Table 1; Figure 1). From 8 trials complete datasets were analysed [14–21]. From two studies, safety data were not available for analysis [13, 22]. All studies included had been approved by ethical committees.

**Table 1:** Summary of studies included in the meta-analysis

Author	Regimens	Elderly patients/total patients	Primary endpoint	Secondary endpoints	Reference
Borner 2008	Capecitabine + oxaliplatin Capecitabine + oxaliplatin + cetuximab	12/37 4/37	ORR	OS, TTP, TTF	13
Diaz-Rubio 2012	XELOX + bevacizumab → maintenance bevacizumab + XELOX or maintenance bevacizumab	70/239 0/241	PFS	OS, ORR, TTR, DoR, safety	14
Dotan 2012	Capecitabine + oxaliplatin + cetuximab + bevacizumab Capecitabine + oxaliplatin + cetuximab	0/12 1/11	ORR	OS, TTP	15
Feliu 2010	Capecitabine + bevacizumab	59/59	ORR	PFS, OS, safety	16
Okita 2012	FOLFOX6 + bevacizumab	7/50	ORR	TTF, PFS, OS, AEs, neurotoxicity	17
Souglakos 2012	CAPIRI + bevacizumab FOLFIRI + bevacizumab	63/167 55/166	PFS	OS, ORR, safety	18
Tol 2009	Capecitabine + oxaliplatin + bevacizumab Capecitabine + oxaliplatin + bevacizumab + cetuximab	81/378 0/377	PFS	OS, ORR, safety, QoL, KRAS status/EGFR	19
Arkenau 2008	CAPOX FUFOX	75/241 64/233	PFS	ORR, OS, TTF	20
Koopman 2007	Capecitabine → irinotecan → capecitabine + oxaliplatin Capecitabine + irinotecan → capecitabine + oxaliplatin	26/401 38/402	OS	PFS, OS, toxicity, QoL	21
Tournigand 2004	FOLFIRI → FOLFOX6 FOLFOX6 → FOLFIRI	19/109 31/111	OS	PFS, ORR, safety	22

DoR, duration of response; PFS, progression-free survival; QoL, quality of life; ORR, objective response rate; TTF, time to treatment failure; TTP, time to progression; TTR, time to response

### Outcomes

The 10 studies from which we received primary data included a total of 3271 patients, of which 604 patients (18%; ranging from between 1 and 139 patients per study) were 70 years or older and were included in the dataset (Table 1). Across all patients included in the meta-analysis, 375 (62.6%) were male and the median age was 73 years (range 70–88 years). There was no significant difference (*p* > 0.20) in patients’ age distribution between those treated with or without bevacizumab (Supplementary Figure 1). In total, 335 patients were treated with a bevacizumab-based first-line regimen and 264 patients were treated with chemotherapy only. Since only 5 patients were treated with a cetuximab-based first-line regimen, this group was excluded from further analysis.

### Primary analysis

**Figure 2:**
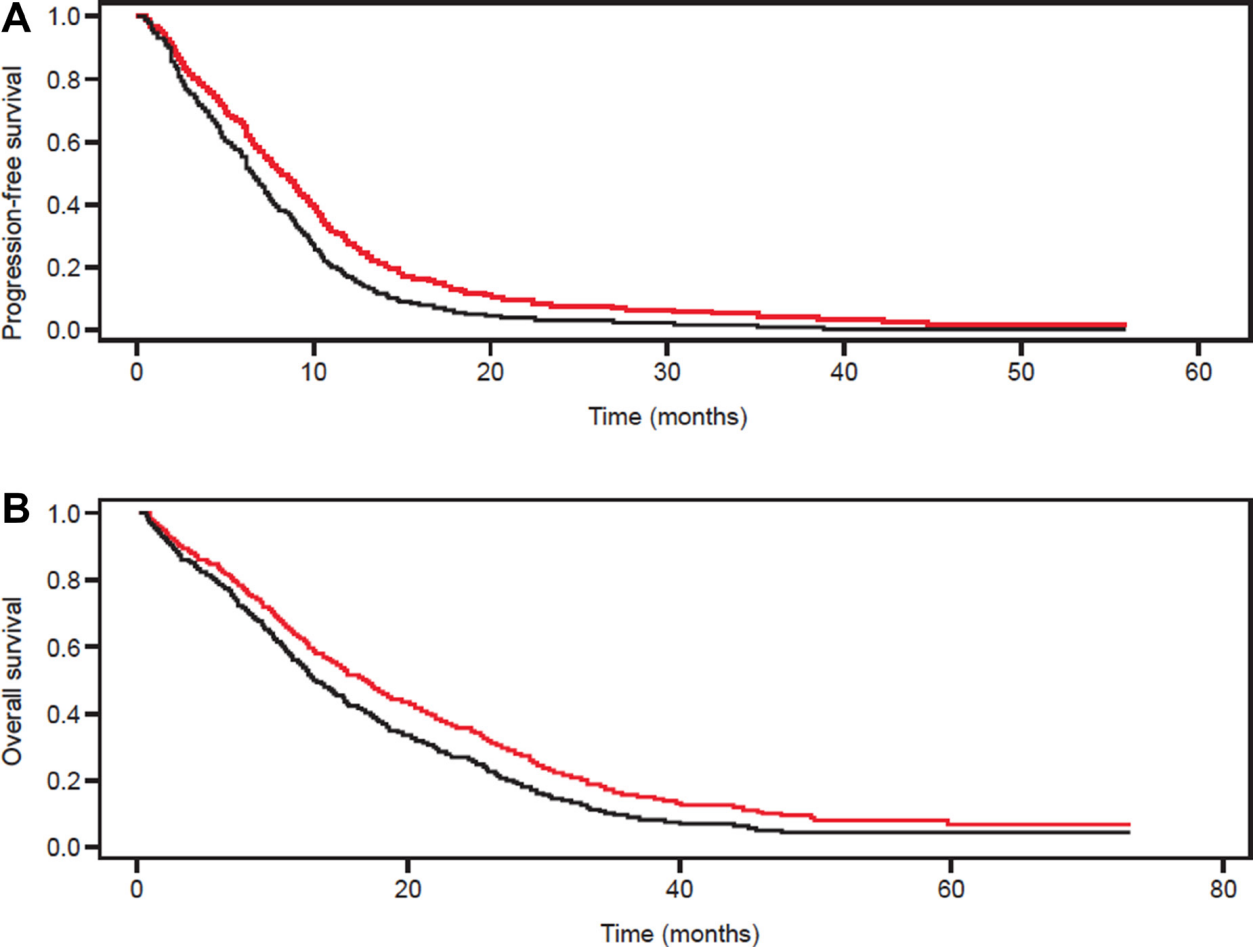
(**A**) Progression-free survival and (**B**) overall survival in patients ≥ 70 years of age with mCRC treated with standard chemotherapy with (red lines) or without (black lines) bevacizumab in the single study populations. Displayed is the summary estimation from a Cox regression with frailty approach. The median PFS was 8.2 vs. 6.5 months and the median OS was 16.7 vs. 13.0 months in patients treated with and without bevacizumab, respectively.

**Figure 3:**
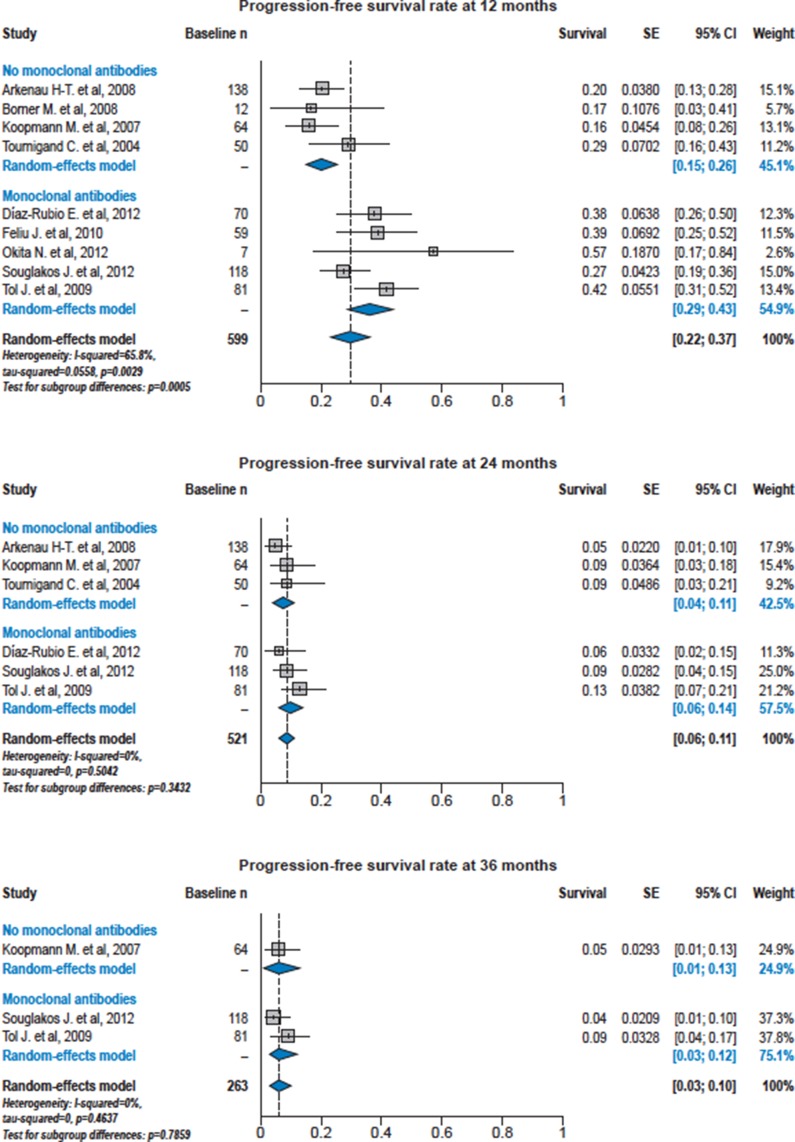
Forest plots of progression-free survival rates at 12, 24 and 36 months from the individual studies in patients ≥ 70 years of age treated with or without bevacizumab For each study arm, survival rates and standard error are derived by single nonparametric Kaplan-Meier estimates.

**Figure 4:**
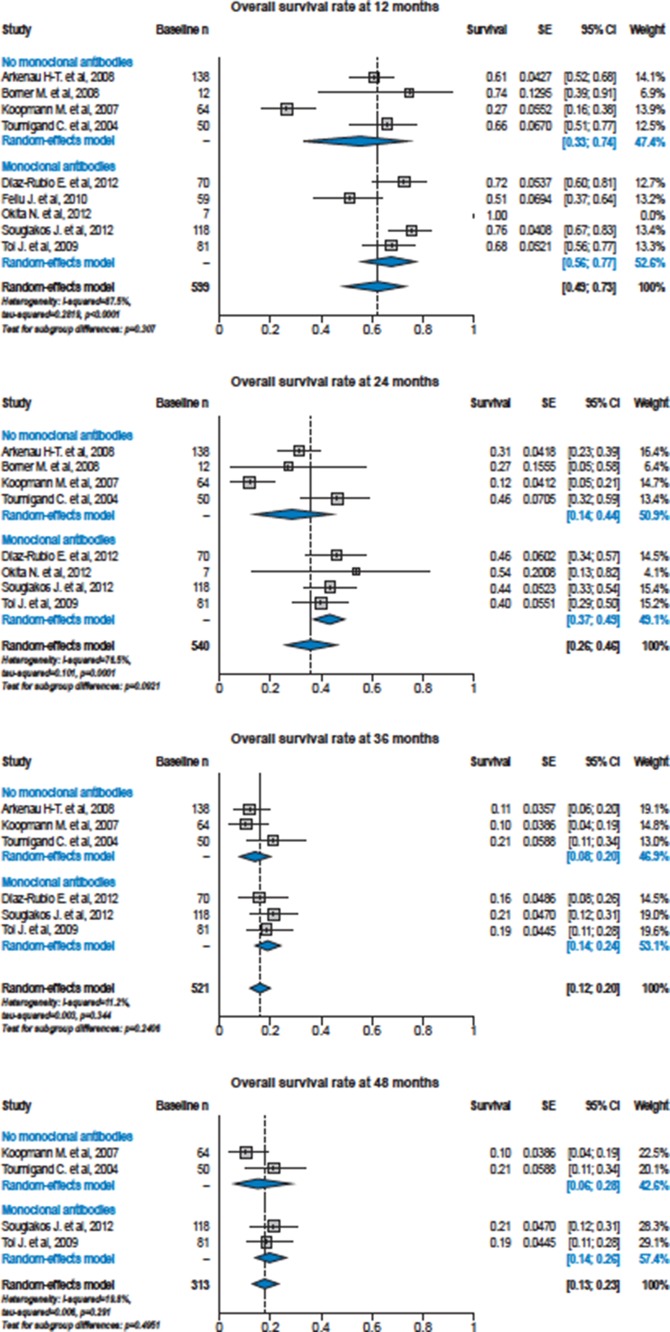
Forest plots of overall survival rates at 12, 24, 36 and 48 months from the individual studies in patients ≥ 70 years of age treated with or without bevacizumab For each study arm, survival rates and standard error are derived by single nonparametric Kaplan-Meier estimates.

Median follow-up time ranged from 7.4 months to 23.4 months in the single studies. For the primary outcomes of PFS and OS, there was a high degree of heterogeneity between the sites; Figure 2 shows the overall summary curves derived from Cox regression for PFS and OS for patients ≥ 70 years of age treated with or without bevacizumab and also displays the Kaplan-Meier survival curves from the individual study arms. Both PFS and OS were significantly increased in patients receiving chemotherapy with bevacizumab (PFS: HR 1.39, 95% CI 1.14–1.70; *p* = 0.0014; OS: HR 1.29, 95% CI 1.04–1.60; *p* = 0.019). The median PFS was 8.2 vs. 6.5 months, the median OS was 16.7 vs. 13.0 months in patients treated with and without bevacizumab, respectively. For both PFS and OS, variance of the random effect terms was 0.004. Forest plots of PFS and OS rates at 12, 24, 36 and 48 months from the individual studies in patients ≥ 70 years of age treated with or without bevacizumab show variation with and between studies (Figures 3 and 4).

Assessment of potential risk of selection or publication bias across the studies also used PFS and OS rates at 12, 24, 36 and 48 months. Funnel plots for PFS and OS rates at 12 months showed no signs of asymmetry (*p* = 0.20 and *p* > 0.20, respectively; see Supplementary Figure 2), which would indicate the absence of selection bias. A sensitivity analysis was also performed to determine whether the results in controlled but non-randomised studies differed from those of randomised studies – this correction for study type was not statistically significant and the HR of bevacizumab treatment changed only marginally.

### Additional analyses

**Figure 5:**
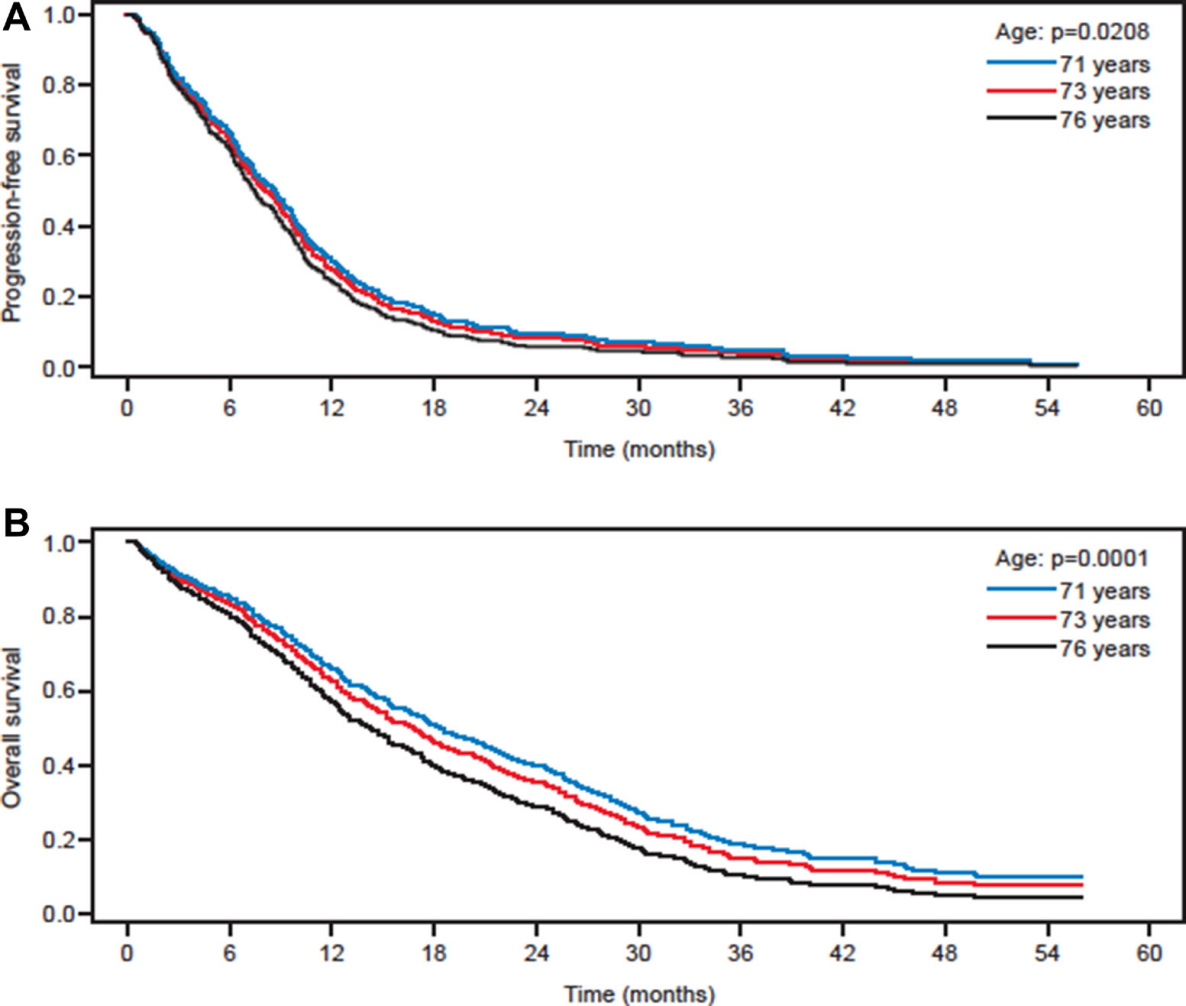
Effect of age in all patients on: (**A**) progression-free and (**B**) overall survival based on a Cox regression curve with age as quantitative covariate in patients ≥70 years of age with mCRC. The exemplary values of predicted survival curves at 71, 73 and 76 years of age correspond to the first quartile, median age and third quartile, respectively.

Further analyses were performed evaluating the influence of age, sex and a number of other factors included in the dataset on OS and PFS with the corresponding Cox regression models including these factors or covariates as well as a frailty term to account for heterogeneity. The strongest association for both PFS and OS was found to be age (*p* = 0.0210 and *p* < 0.0001, respectively; Figure 5), bleedings of grade 3 or 4 (*p* = 0.0112 and *p* = 0.0005, respectively) and hand–foot syndrome (*p* = 0.007 and *p* = 0.025, respectively). Multivariate analyses confirmed that treatment with bevacizumab significantly increased PFS and OS compared with chemotherapy treatment only (*p* = 0.0048 and *p* = 0.0016, respectively; Supplementary Table 1).

Comparison of frequencies of markers of toxicity between study arms of patients treated with and without antibodies found that there was no significant difference of toxicities in patients treated with bevacizumab than without (Table 2; Supplementary Figure 3).

**Table 2:** Incidence adverse events (CTC grade ≥ 3) in patients ≥ 70 years of age with mCRC treated with or without bevacizumab

Adverse event, % (95% CI)	Bevacizumab	No monoclonal antibody	*P*-value^b^
Hypertension	6 (2–15)	1 (0–5)	0.0766
Nausea and vomiting	5 (2–13)	14 (10–19)	0.0591
Allergic reaction	3 (1–7)	1 (0–5)	*p* > 0.20
Hand–foot syndrome	9 (4–20)	3 (1–15)	*p* > 0.20
Dermatological changes^a^	1 (0–4)	1 (0–5)	*p* > 0.20
Gastrointestinal perforation	2 (1–5)	1 (0–5)	*p* > 0.20
Electrolyte imbalance	2 (1–4)	1 (0–5)	*p* > 0.20
Fracture	2 (1–4)	1 (0–5)	*p* > 0.20
Urological AEs^b^	2 (1–5)	1 (0–5)	*p* > 0.20
Syncope	2 (1–5)	1 (0–5)	*p* > 0.20
Diarrhoea	11 (5–21)	6 (1–33)	*p* > 0.20
Pain^c^	4 (2–8)	3 (2–7)	*p* > 0.20
Thrombosis, embolism, phlebitis	5 (2–13)	3 (1–18)	*p* > 0.20
Dehydration	2 (1–5)	2 (1–7)	*p* > 0.20
Myelosuppression	9 (3–24)	6 (1–24)	*p* > 0.20
Infection^d^	6 (3–13)	9 (6–13)	*p* > 0.20
Weight loss/loss of appetite	5 (2–11)	4 (1–21)	*p* > 0.20
Fatigue	8 (2–25)	5 (1–29)	*p* > 0.20
Neuropathy	6 (2–20)	5 (1–33)	*p* > 0.20
Gastrointestinal AEs^e^	4 (2–9)	3 (1–18)	*p* > 0.20
Bleeding	1 (0–3)	3 (1–14)	*p* > 0.20
Dyspnoea	3 (1–6)	3 1–8)	*p* > 0.20
Febrile neutropenia	2 (1–4)	3 (0–23)	*p* > 0.20
Cardiac events^f^	3 (2–7)	4 (2–8)	*p* > 0.20

^a^Acne, nail changes, other (not specified by authors of original study/publication).

^b^Incontinence, urinary retention, elevated creatinine.

^c^Musculoskeletal, visceral, tumour pain.

^d^Includes mucositis, sepsis.

^e^Xerostomia, constipation, ileus, malabsorption, ulcer, other colon events (not specified by authors of original study/publication).

^f^Ischaemia, transient ischaemic event, angina pectoris, supraventricular tachycardia, other (not specified by authors of original study/publication). The events are sorted by descending p-value.

## DISCUSSION

Elderly patients are often underrepresented in oncological clinical trials. However, the majority of colorectal cancers arise in patients older than 70 years [1, 2]. As the population at least in western countries ages, the term “elderly” needs to be better defined, since it is evident that there is a wide range in the performance status of different patients of the same age. Geriatric assessments are a tool to evaluate and manage functional and cognitive impairments in elderly oncologic patients [28] and should be implemented in daily practise as well as in clinical trials to better distinguish between “fit” and “frail”.

The aim of the study was the assessment of both PFS and OS in patients ≥ 70 years of age treated with chemotherapy with or without a monoclonal antibody. More recently, primary tumor location, RAS and BRAF status were established as important prognostic factors for colorectal cancer patients [28, 29, 30, 31]. However, since our data set was based on studies published until April 2013, we did not address these prognostic factors in our analysis.

The results of this meta-analysis show that both PFS and OS were significantly increased in elderly patients with mCRC receiving chemotherapy plus bevacizumab compared with those receiving chemotherapy alone (Figure 2A, 2B), and there would appear to be a lack of selection bias in this finding. As would be anticipated in an elderly population, PFS and OS were influenced by age with both outcomes being greater in the ‘younger’ population (Figure 5); the population that would be anticipated to have prolonged survival in a disease-free state. A finding that was observed in the BRiTE observational cohort study, in which patients ≥ 65 years of age and receiving chemotherapy plus bevacizumab had similar PFS as those patients < 65 years and receiving the same treatment while, as expected, OS lessened with increased age [23].

The addition of bevacizumab to a chemotherapy regimen was well tolerated in this elderly population, with only a trend towards significance for hypertension (Table 2) in patients treated with bevacizumab; the incidence of all other adverse events was comparable with those receiving chemotherapy alone. Hence, the argument that greater toxicity is observed in elderly patients receiving bevacizumab is not supported by the findings of this meta-analysis.

The efficacy and tolerability results observed in this meta-analysis are in agreement with findings from the phase III AVEX trial, which reported a clinically significant benefit of adding bevacizumab to low doses of capecitabine (2000 mg/m^2^/day) in patients aged ≥ 70 years not deemed suitable for treatment with chemotherapy doublets. Patients with a median age of 76–77 years gained a 4-month PFS benefit (HR: 0.53, 95% CI: 0.41–0.69; *p* < 0.001) and a clinically, but not statistically, significant OS benefit of 3.9 months (HR: 0.79, 95% CI: 0.57–1.09; *p* = 0.182) with bevacizumab plus capecitabine versus capecitabine alone [7]. Recently, data from a single-arm Japanese phase 2 trial, including 55 patients with mCRC, were published, demonstrating that the oral fluoropyrimidine UFT combined with biweekly bevacizumab is a tolerable and effective treatment option for elderly patients [9]. Similarly, a pooled analysis of four randomised clinical studies comparing elderly with younger mCRC patients showed that the addition of bevacizumab to chemotherapy provided comparable PFS and OS benefits in medically fit older patients [24]. Furthermore, in the randomised AGITG MAX study, the improvement in PFS observed when bevacizumab was added to the existing chemotherapy regimen was similar in those patients ≥ 75 years of age compared with younger patients [25]. However, not all studies evaluating the effect of age on mCRC treatment outcome with chemotherapy plus a monoclonal antibody have reported similar outcomes in the two age groups. For example, in a US observational cohort study (Avastin^®^ Registry: Investigation of Effectiveness and Safety; ARIES), there were slight reductions in PFS (10.3 vs. 9.9 months) and OS (25.1 vs. 19.6 months) in mCRC patients ≥ 70 years compared with those < 70 years of age receiving bevacizumab and chemotherapy in the first-line setting; interestingly though, PFS (7.9 vs. 7.9 months) and OS (18.7 vs. 17.2 months) did not differ in the second-line setting when comparing the two age groups [26, 27].

There are a number of limitations of this meta-analysis at both the study and outcome level that need to be addressed. There is the risk of bias in the findings, with incomplete retrieval of clinical data from all of the identified research generating a potential bias towards those studies that have shared their data. It is also feasible that as elderly patients increase in age they might also have a poorer general prognosis and certainly have a higher probability of non-tumor-related death, which could confound the results somewhat. The primary goal of the study was to provide sufficient data both for the use of bevacizumab as well as for the use of either cetuximab or panitumumab in combination with first-line chemotherapy in elderly patients. However, we did not receive data from a sufficient number of studies/patients receiving one of these anti-EGF-receptor antibodies in combination with first-line chemotherapy. Therefore, further analyses on the use of anti-EGF-receptor based regimen in elderly patients with mCRC are necessary.

In conclusion, elderly patients with mCRC are frequently underrepresented in clinical trials. Published phase II and III clinical trials that evaluated monoclonal antibodies plus chemotherapy did not include subgroup analyses of elderly patients [32, 33, 34] or showed an unfavourable outcome of patients older than 75 years for FOLFOX plus anti-EGF-receptor antibodies [35]. A pooled analysis of studies with bevacizumab suggested a benefit for patients older than 65 years, however only PFS was superior [36]. In real-life the majority of patients with mCRC are older than 65 years and undertreatment in this large cohort of patients just based on age must be avoided. While each patient should be considered on an individual basis, the results of this meta-analysis suggest that the addition of bevacizumab to a first-line chemotherapy regimen improves clinical outcome in elderly patients while remaining well tolerated. Further studies are warranted with recruitment of patients of an appropriate age in clinical trials that better reflects incidence rates of the disease in the general population.

## MATERIALS AND METHODS

### Protocol and registration

This individual patient data based meta-analysis was planned in advance; however, the protocol has not been published and the meta-analysis has not been registered.

### Inclusion and exclusion criteria

Inclusion criteria: controlled, comparator phase II or III studies that included patients aged ≥ 70 years, with Eastern Cooperative Oncology Group Performance Status (ECOG PS) ≤ 1 and previously untreated mCRC; patients received standard first-line chemotherapy with or without a monoclonal antibody (bevacizumab, cetuximab, panitumumab [cetuximab and panitumumab only if the KRAS status was known at study start]).

Exclusion criteria: retrospective cohort studies, observational studies, previous treatment for liver metastases, off-label therapies, second-line or subsequent regimens, adjuvant therapy, combination with multiple antibodies in all treatment arms.

### Search strategy and data extraction

PubMed and the Cochrane Library were searched using the following terms: (colorectal [title] AND cancer [title] AND bevacizumab [title]); (colorectal [title] AND cancer [title] AND cetuximab [title]); and (colorectal [title] AND cancer [title] AND panitumumab [title]). The Cochrane Library was searched using the following terms: (colorectal [Title, Abstract, Keywords] AND cancer [Title, Abstract, Keywords] AND bevacizumab [Title, Abstract, Keywords]); (colorectal [Title, Abstract, Keywords] AND cancer [Title, Abstract, Keywords] AND cetuximab [Title, Abstract, Keywords]); and (colorectal [Title, Abstract, Keywords] AND cancer [Title, Abstract, Keywords] AND panitumumab [Title, Abstract, Keywords]).

The PubMed and the Cochrane Library searches were performed on 29 April 2013 and only studies published up until this date were included in this meta-analysis. Abstract databases and congress websites were not searched or included in this analysis.

The search results were screened for relevance by reading the titles and abstracts, and any duplicates were removed; potentially relevant articles were read by two independent reviewers to identify those studies that satisfied the inclusion and exclusion criteria.

### Data acquisition

Authors of each of the identified articles were contacted to request information on patients ≥ 70 years of age in the respective studies. The requested information included data on progression-free survival (PFS) and overall survival (OS), together with individual patient data on treatment regimes, age and sex. In addition to this, data were also collected on any potential signs of toxicity such as myelosuppression, electrolyte imbalance, fatigue, bleeding, nausea and vomiting, gastrointestinal adverse events (AEs; xerostomia, constipation, ileus, malabsorption, ulcers, and other colon event), weight loss/loss of appetite, dehydration, febrile neutropenia due to myelosuppression, infection (including mucositis, sepsis), cardiovascular events (ischaemia, transient ischaemic event, other), hypertension, urological AEs (incontinence, urinary retention, elevated creatinine), dyspnoea, dermatological changes (acne, nail changes, other), hand-foot syndrome, thrombosis/embolism/phlebitis, allergic reaction, neuropathy, pain (muscloskeletal, visceral, tumor pain), fracture and other signs.

### Statistical analysis

#### Synthesis of results

The primary aim was the assessment of both PFS and OS in patients ≥ 70 years of age treated with chemotherapy with or without a monoclonal antibody. As for potential confounding factors, this comparison was done using a Cox proportional hazards model to account for the heterogeneity between studies with a gamma-frailty modelling approach with one degree of freedom. Survival curves are compared with Kaplan-Meier curves in the single study populations to illustrate heterogeneity.

### Risk of bias across studies

Risk of selection and publication bias was assessed by funnel plots comparing correlations of PFS and OS for each treatment group with sample size.

### Additional analyses

Besides the primary analysis of survival data for comparison of the treatment groups, potential toxicity markers (as described earlier) and sex (categorical factor) and age (continuous covariate) were analysed by univariate and multivariable analyses using a stepwise backward Cox regression model for PFS and OS including, again, a frailty term. In addition, Forest plots were generated for estimation of PFS and OS at 1, 2, 3 and 4 years using a log-log transformation to estimate confidence intervals and summarise across study sites. Graphical illustrations of the predicted survival curves of univariate Cox regression discriminating between factor groups and showing typical levels for continuous covariates are presented. Overall estimations using Cox regression with a 95% confidence interval (CI) of the hazard ratio (HR) are reported.

The incidences of potential toxicity markers included in the dataset were evaluated for treatment arms with and without monoclonal antibodies. These incidences were summarised by calculating overall proportions using a DerSimonian and Laird random effect model on logit scale. A two-sided Q-test was used to compare incidences in patients treated with and without monoclonal antibodies and this analysis was illustrated using Forest plots.

In addition to these main analyses, standard descriptive analyses (median, range, boxplots, frequencies) were used to illustrate the clinical characteristics of the dataset.

All statistical tests were two-sided and used a significance level of a = 5%. The statistical analysis was performed with R 3.2.3 software (R Foundation for Statistical Computing, Vienna, Austria) using, especially, the survival and meta packages.

## SUPPLEMENTARY MATERIALS FIGURES AND TABLE



## Abbreviations

mCRC: metastatic colorectal cancer
OS: overall survival
PFS: progression-free survival.
