# Supplementary material for: Bevacizumab-based first-line chemotherapy in elderly patients with metastatic colorectal cancer: an individual patient data based meta-analysis
Source: Oncotarget. 2017 Dec 20;9(12):10272–83. doi: 10.18632/oncotarget.23475 (PMC5828201; doi:10.18632/oncotarget.23475)
Supplement: Supplementary file 1 [file oncotarget-09-10272-s001.pdf]

Bevacizumab-based first-line chemotherapy in elderly patients with metastatic colorectal cancer: an individual patient data based meta-analysis

SUPPLEMENTARY MATERIALS

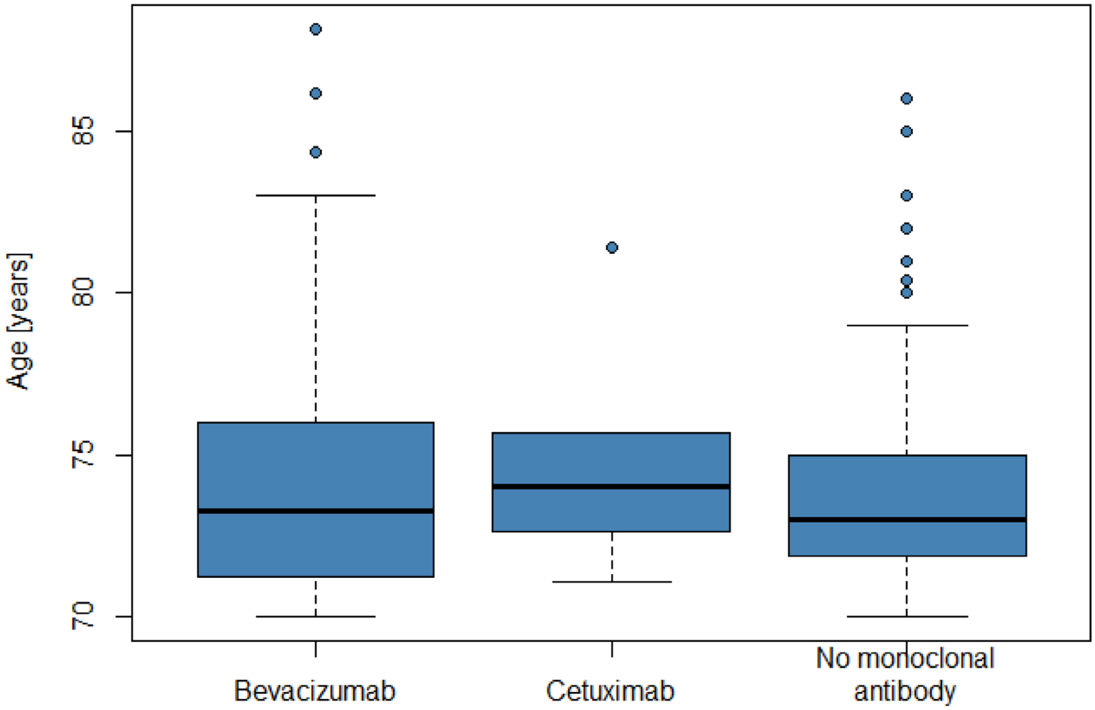

Supplementary Figure 1: Age distribution of patients ≥ 70 years of age with mCRC treated with or without bevacizumab.

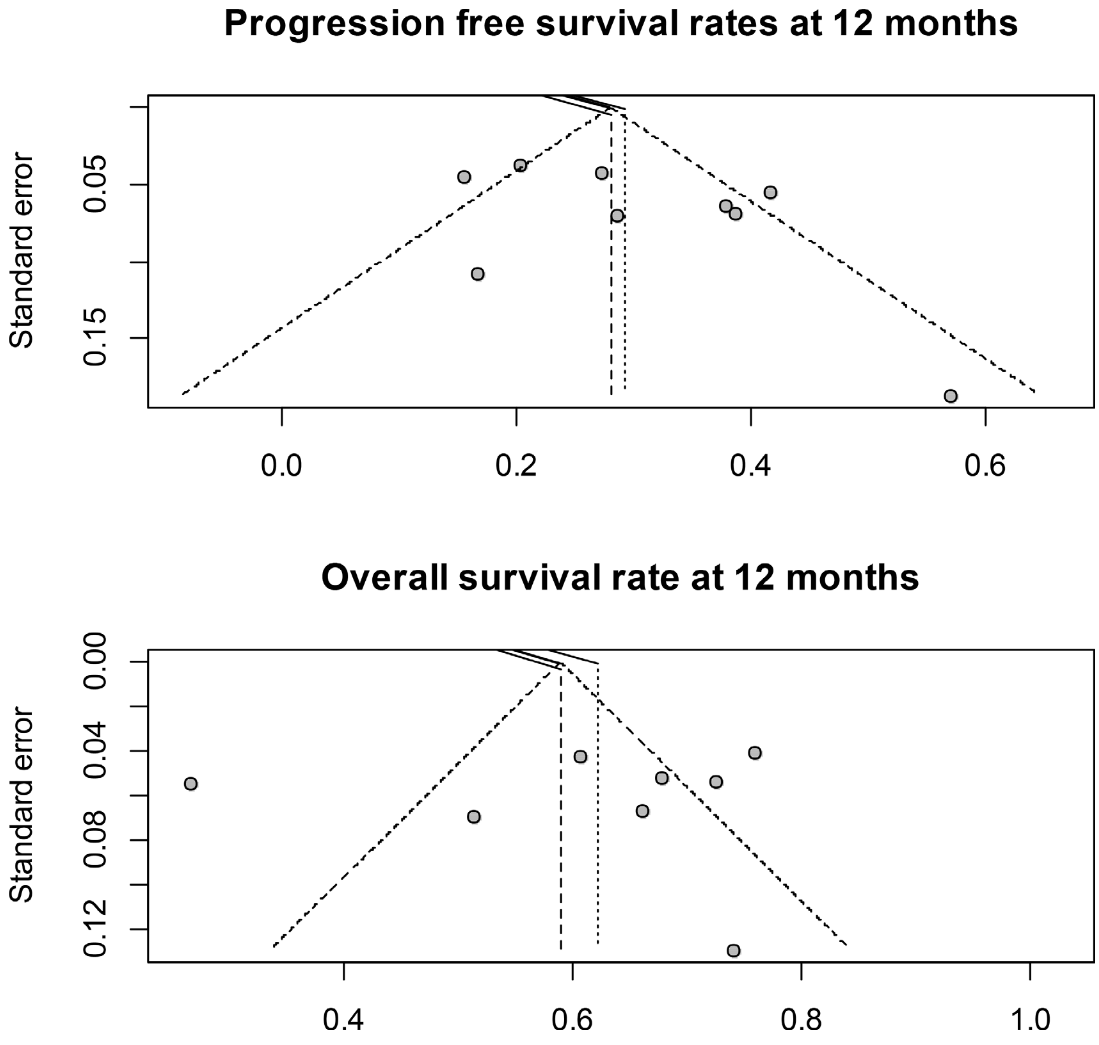

Supplementary Figure 2: Funnel plots for progression-free survival (upper panel) and overall survival (lower panel) at 12 months showing there are no indications of selection bias.

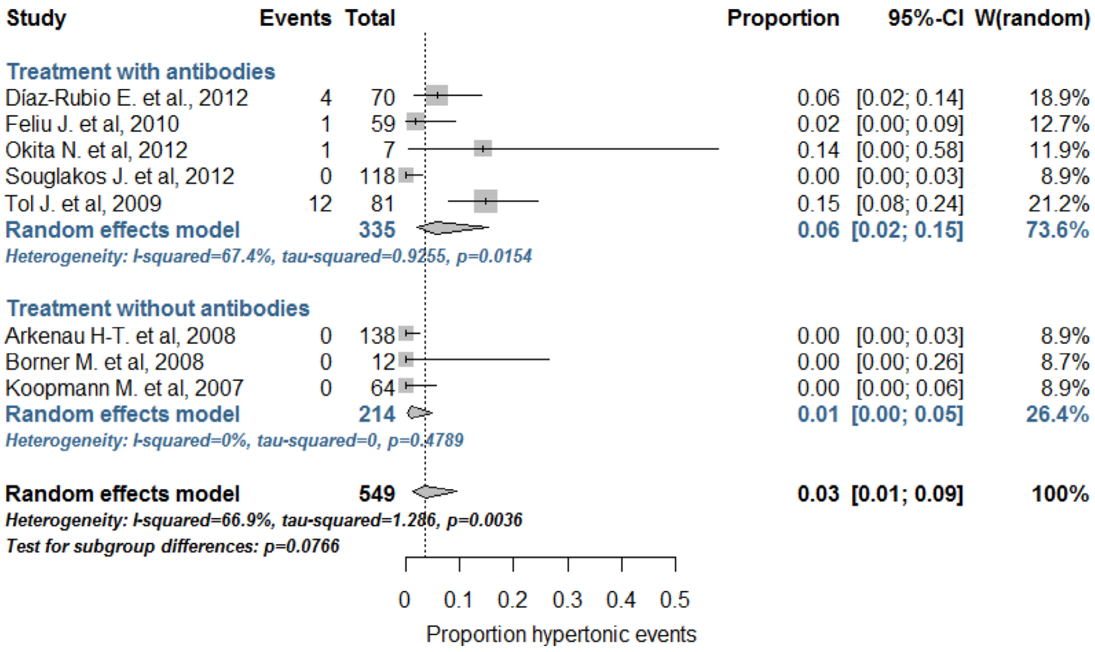

Supplementary Figure 3: Effect of bevacizumab on the incidence of hypertension in patients ≥ 70 years of age with mCRC.

Supplementary Table 1: Multivariate cox regression analysis of various demographic and clinical characteristics on PFS and OS in in patients ≥ 70 years of age with mCRC

Variable	HR [95% CI]	*P*-value
PFS		
No monoclonal antibody use	1.40 [1.12–1.75]	0.0048
Age [per year]	1.03 [1.001–1.06]	0.0105
Neuropathy:		
Grade 0–2	Reference	
Grade 3	0.65 [0.47–0.91]	
Grade 4	1.00 [0.37–2.71]	0.0105
Dehydration:		
Grade 0–2	Reference	
Grade 3–4	3.00 [1.32–6.78]	0.0184
Bleeding grade 3-4	3.34 [1.36–8.21]	0.0199
Hand-foot syndrome:		
Grade 0–2	Reference	
Grade 3	0.64 [0.44–0.94]	0.0276
OS		
No monoclonal antibody use	1.41 [1.10–1.80]	0.0016
Age [per year]	1.06 [1.03–1.09]	< 0.0001
Bleeding grade 3-4	4.83 [1.85–12.65]	0.0012
Dyspnoea:		
Grade 0–2	Reference	
Grade 3	3.319 [1.12–9.84]	
Grade 4–5	6.17 [2.05–18.37]	0.0012
Dehydration:		
Grade 0–2	Reference	
Grade 3–4	1.70 [1.68–12.66]	0.0221
Neuropathy:		
Grade 0–2	Reference	
Grade 3	0.61 [0.42–0.88]	
Grade 4	0.99 [0.39–2.54]	0.0036
Fracture:		
Grade 0–2	Reference	
Grade 3	0.14 [0.02–1.07]	0.038
Cardiac events	1.56 [0.86–2.83]	0.0472
